# Genotype-phenotype correlation analysis of *MYO15A* variants in autosomal recessive non-syndromic hearing loss

**DOI:** 10.1186/s12881-019-0790-2

**Published:** 2019-04-05

**Authors:** Jing Zhang, Jing Guan, Hongyang Wang, Linwei Yin, Dayong Wang, Lidong Zhao, Huifang Zhou, Qiuju Wang

**Affiliations:** 1 Chinese PLA Institute of Otolaryngology, Chinese PLA General Hospital, Medical School of Chinese PLA, 28 Fuxing Road, Beijing, 100853 China; 2 Department of Otolaryngology of Tianjin Medical University General Hospital, Tianjin, 300052 China; 3 BGI-Shenzhen, Shenzhen, 518120 China

**Keywords:** *MYO15A*, *DFNB3*, Phenotype, Deafness

## Abstract

**Background:**

*MYO15A* variants are responsible for human non-syndromic autosomal recessive deafness (*DFNB3*). The majority of *MYO15A* variants are associated with a congenital severe-to-profound hearing loss phenotype, except for *MYO15A* variants in exon 2, which cause a milder auditory phenotype, suggesting a genotype-phenotype correlation of *MYO15A*. However, *MYO15A* variants not in exon 2 related to a milder phenotype have also been reported, indicating that the genotype-phenotype correlation of *MYO15A* is complicated. This study aimed to provide more cases of *MYO15A* variation with diverse phenotypes to analyse this complex correlation.

**Methods:**

Fifteen Chinese autosomal recessive non-syndromic hearing loss (ARNSHL) individuals with *MYO15A* variants (8 males and 7 females) from 14 unrelated families, identified by targeted gene capture of 127 known candidate deafness genes, were recruited. Additionally, we conducted a review of the literature to further analyses all reported *MYO15A* genotype-phenotype relationships worldwide.

**Results:**

We identified 16 novel variants and 12 reported pathogenic *MYO15A* variants in 15 patients, two of which presented with a milder phenotype. Interestingly, one of these cases carried two reported pathogenic variants in exon 2, while the other carried two novel variants not in exon 2. Based on our literature review, *MYO15A* genotype-phenotype correlation analysis showed that almost all domains were reported to be correlated with a milder phenotype. However, variants in the N-terminal domain were more likely to cause a milder phenotype. Using next-generation sequencing (NGS), we also found that the number of known *MYO15A* variants with milder phenotypes in Southeast Asia has increased in recent years.

**Conclusion:**

Our work extended the *MYO15A* variant spectrum, enriched our knowledge of auditory phenotypes, and tried to explore the genotype-phenotype correlation in different populations in order to investigate the cause of the complex *MYO15A* genotype-phenotype correlation.

**Electronic supplementary material:**

The online version of this article (10.1186/s12881-019-0790-2) contains supplementary material, which is available to authorized users.

## Background

Sensorineural hearing loss (SNHL) is one of the most common sensory defects, affecting approximately 466 million people worldwide, 34 million of whom are children (http://www.who.int/). More than 50% of congenital hearing impairment cases are attributable to genetic factors, with more than 100 syndromic and non-syndromic genes known to date. Non-syndromic hearing loss (NSHL) is further categorized as autosomal dominant, autosomal recessive, X-linked, Y-linked or mitochondrial hearing loss. Autosomal recessive non-syndromic hearing loss (ARNSHL) accounts for up to 70% of NSHL. As of Jan 2019, more than 70 genes have been reported to be responsible for ARNSHL (http://hereditaryhearingloss.org/). Variants of *GJB2*, *SLC26A4*, *MYO15A*, *OTOF, CDH23*, *MYO7A, TMC1, HGF* and *CIB* are worldwide major contributors to ARNSHL [1], and myosin 15A (*MYO15A*, MIM: 602666) gene variants are the third or fourth most frequent causes of ARNSHL [2]. According to previous reports, the prevalence of *MYO15A* variants was 3~9.9% (19/557) among Turkish ARNSHL patients, 5.7% among Pakistani patients [1], 5.7% among Iranian patients [3], 2.71% among Korean patients, 1.67% among Japanese patients (10/600) and 2.6% among Chinese patients.

Encoded by *MYO15A*, myosin XVa is a member of the unconventional myosin superfamily and plays an indispensable role in the graded elongation of stereocilia and actin organization in hair cells of the inner ear, which are essential for normal hearing function [4]. As a result, *MYO15A* variants are responsible for non-syndromic autosomal recessive deafness (DFNB3, OMIM 600316) in humans, whereas variants of homologous *Myo15* genes lead to deafness and vestibular dysfunction in mice (*shaker-2*) [5–8].

Initially, the majority of *MYO15A* variants were documented in patients with severe to profound congenital sensorineural deafness phenotypes from consanguineous families in the Middle East and Southeast Asia. However, with the recent development of next-generation sequencing (NGS), the reported number of *MYO15A* variants has sharply increased in other countries and regions, such as East Asia and Europe. Contrary to previous reported severe auditory phenotypes, partial deafness with residual hearing at low frequencies was reported, always associated with pathologic variants in exon 2 affecting the encoding of the N-terminal domain. Interestingly, recent studies have reported that some variants of *MYO15A* affecting the function of other domains, which were expected to bring about congenital severe to profound hearing loss, also were observed to result in various milder auditory phenotypes [9, 10].

In the present study, we reported 15 Chinese *MYO15A* variant cases from 14 unrelated ARNSHL families and identified 16 novel variants and 12 *MYO15A* variants in total by targeted gene capture and high-throughput sequencing. Additionally, we analysed all reported *MYO15A* genotype-phenotype relationships worldwide through a review of the literature. Our work extended the *MYO15A* variant spectrum, enriched our knowledge of auditory phenotypes, and tried to explore the genotype-phenotype correlation in different populations in order to investigate the cause of the complex *MYO15A* genotype-phenotype correlation.

## Methods

### Participants

A total of 15 Chinese ARNSHL individuals, ethnic Han, with *MYO15A* variants (8 males and 7 females) from 14 unrelated families, were recruited by the Department of Otolaryngology, Head and Neck Surgery of the Chinese PLA General Hospital. 127 known candidate deafness genes identifications were performed with targeted gene capture.

### Clinical evaluations

For auditory assessment in all subjects, otoscopy, tympanometry, acoustic reflex thresholds, distortion product otoacoustic emission (DPOAE), and pure-tone audiometry following standard protocols were included. (Behavioural observation audiometry, visual reinforcement audiometry or play audiometry for young children.) Auditory brainstem response (ABR) and auditory steady state response (ASSR) were applied if the above subjective audiometric assessments could not be performed in infants. Family history and clinical questionnaires were available from all subjects or relatives.

The definition for severity of hearing impairment, according to pure-tone audiometry (PTA) of the better ear, was made based on the average hearing threshold level at four frequencies (500, 1000, 2000 and 4000 Hz) of air conduction. 26–40 dB HL were considered to be mild hearing loss; 41–55 dB HL, moderate hearing loss; 56–70 dB, moderately severe hearing loss; 71–90 dB HL, severe hearing loss; > 90 dB HL, profound hearing loss. The occurrence of hearing loss was categorized as prelingual (≤6 years) or post-lingual (> 6 years).

### Targeted gene capture and high throughput sequencing

Blood DNA kit (Tiangen Biotech, Beijing, China) was used to extract Genomic DNA of all participants from peripheral blood. Targeted gene capture and high-throughput sequencing were then applied as reported thoroughly in a previous study [11]. Briefly, sequences of exons, splicing sites and flanking intron of 127 known deafness genes were captured simultaneously using a targeted genomic enrichment platform (Additional file 1: Table S1). Then the DNA library was established after performing sequencing of the target region in subjects. Targeted genes were amplified by PCR using universal primers, followed by sequencing of the PCR products through Illumina HiSeq 2000 (San Diego, California, USA). Burrows-Wheeler Aligner (BWA) (http://bio-bwa.sourceforge.net/) was applied to align reads to the NCBI37/hg19 assembly. SNPs, inserts and deletions were detected by Genome Analysis Toolkit (GATK) software and SAM tools (http://samtools.sourceforge.net/).

### Mutation analysis and control screening

Potentially pathogenic variants including missense, nonsense, splice-site or frameshift variants with an allele frequency less than 0.01 were identified using the Genome Aggregation Database, the Exome Aggregation Consortium and 1000 Genomes databases. Simultaneous detection of point mutations, micro-indels, and duplications (< 20 bp) could be achieved. Pathogenicity of each missense variant was predicted using SIFT (http://sift.jcvi.org), PolyPhen-2 (http://genetics.bwh.harvard.edu/pph2/) and Mutation Taster analyses (http://www.mutationtaster.org), while, GERP++ software (http://mendel.stanford.edu/sidowlab/downloads/gerp/index.html) and PhyloP (http: //compgen.bscb.cornell.edu/phast/) were performed to estimate the evolutionary conservation of the amino acid sequence. Genotyping for candidate pathogenic variants of all family members were underwent with Sanger sequencing. A PE9700 thermocycler (Applied Biosystems, Foster City, CA) was obtained for PCR, and then sequence analysis was carried out using an ABI 3730 Genetic Analyzer on affected cases and normal controls to determine if potential pathogenic mutations in the gene were co-segregating with the disease phenotype of these families. Further, aligning sequences were performed with the NCBI Reference Sequence and DNAStar software (DNASTAR, Madison, WI) to identify variants and polymorphisms. Finally, we selected 200 blood DNA samples from Chinese Han individuals with normal hearing as ethnically matched controls.

### Literature review of genotype-phenotype correlation of *MYO15A* variants

Relevant researches were searched through PubMed using the MeSH terms “*MYO15A*” and “*DFNB3*” up until Jan 30, 2019. Inclusion criteria: 1) original articles and 2) reports of *MYO15A* variants with ARNSHL in humans.

## Results

### Identification of *MYO15A* variants

We identified a total of 28 potentially pathogenic variants of *MYO15A* in 15 NSHL patients from 14 families, only one of which was homozygous and 14 were compound heterozygotes (Additional file 2: Figure S1). Among all 28 *MYO15A* variants, 16 variants were novel: 9 missense variants (p.Arg1248Trp, p.Ala1556Thr, p.Ser1583Pro, p.Ala1608Glu, p.Leu1836Pro, p.Pro2160Leu, p.Asp2608Asn, p.Arg2924His, p.Leu3501Glu), 3 splice site variants (c.4597-2A > G, c.6177 + 1G > T, c.7396-1G > A), 3 frameshift variants (p.Gln2571Hisfs*35, p.Asn2678fs, Leu2693Cysfs*45) and one nonsense variant (p.Arg1898*). Coincidentally, two families (family 1,607,486 and 707,757) in our study shared one identical novel variant, c.7396-1G > A. The remaining 12 variants were previously reported: p.Pro286Serfs*15 [12], p.Ser1176Valfs*14 [13], p.Gly1418Arg [14], p.Ser1481Pro [15–17], c.4596 + 1G > A [18], c.5964 + 3G > A [19, 20], p.Arg1993Trp [21], p.Val2266Met [22], p.Arg2775His, p.Trp2931Glyfs*103, p.Phe3420del [20, 21, 23] and p.Ser3474Gly [24]. Co-segregation of these variations with ARNSHL in the families was confirmed using Sanger sequencing. In addition, the allele frequency in the ExAC, 1000 Genomes and gnomAD databases was less than 0.01. Almost all missense variants except p.Arg2924His were predicted to be pathogenic by SIFT, Mutation Taster and PolyPhen-2. These variants were absent in 200 normal Chinese controls (Additional file 3: Table S2). One particular concern was in samples 1,607,545 (p.Pro286Serfs*15, p.Ser1176Valfs*14 and p.Asp2608Asn) and 18,023,134 (c.4597-2A > G, p.Leu2693Cysfs*45 and p.Ser3474Gly), each of which had three *MYO15A* variants (Table 1). In sample 1,607,545, although p.Pro286Serfs*15 and p.Ser1176Valfs*14 have been reported as pathogenic *MYO15A* variants [12, 13], the novel variant p.Asp2608Asn cannot yet be been ruled out because of its extremely low minor allele frequency (MAF) in multiple databases and because of pathogenicity prediction results from missense variant prediction software. Additionally, determining the pathogenicity of the Ser3474Gly variant is quite problematic due to the finding of two homozygotes with an unknown phenotype in the ExAC database and because this variant has been reported as not pathogenic in a Korean *DFNB3* family [24]. Another particular concern is c.5964 + 3G > A, which has been reported three times in the Chinese population and therefore appears to be a common *MYO15A* variant in Chinese NSHL patients [19, 20].

**Table 1:** Summary of clinical data for the 15 ARNSHL patients from 14 unrelated families with *MYO15A* variants

Family ID	Gender	Age of test (year)	Zygosity	Nucleotide Change (NM_016239.3)	Amino Acid Change (NP_057323.3)	Protein Domain	Hearing impairment phenotype			Method of hearing rehabilitation
							Age of onset (year)	Severity (PTA)	Type of audiometry	
139,408	M	23	Hom	c.6479C > T	p.Pro2160Leu	MyTH4 1	Congenital	Profound	Flat	CI(L)
1,507,361	M	3	Het	c.6796G > A	p.Val2266Met	Other	Congenital	Profound	Flat	HA(Bi)
				c.8771G > A	p.Arg2924His	SH3				
1,507,382	M	6	Het	c.4666G > A	p.Ala1556Thr	Motor	Pre-lingual(5 yr) progressive	Severe / residual hearing of low frequencies	Down-sloping	HA(Bi)
				c.6177 + 1G > T	Splice site	Other				
1,606,852	M	2	Het	c.7708_7709insCA	p.Gln2571Hisfs*35	Other	Congenital	Profound	Flat	Nothing
				c.5977C > T	p.Arg1993Trp	Other				
1,607,486–1	F	62	Het	c.4823C > A	p.Ala1608Glu	Motor	Congenital	Profound	Flat	Nothing
				c.7396-1G > A	Splice site	Other				
1,607,486–2	M	66	Het	c.4823C > A	p.Ala1608Glu	Motor	Congenital	Profound	Flat	Nothing
				c.7396-1G > A	Splice site	Other				
1,607,107	F	1	Het	c.5507 T > C	p.Leu1836Pro	Motor	Congenital	Profound	Flat	CI(R)
				c.8324G > A	p.Arg2775His	FERM 1				
1,607,545	F	3	Het	c.855dupT	p.Pro286Serfs*15	N-terminal	Congenital progressive	Severe /residual hearing of low frequencies	Down-sloping	HA(Bi)
				c.3524dupA	p.Ser1176Valfs*14	N-terminal				
				c.7822G > A	p.Asp2608Asn	Other				
1,607,551	M	30	Het	c.4441 T > C	p.Ser1481Pro	Motor	Congenital	Severe	Flat	HA(Bi)
				c.8033_8056del	p.Asn2678fs	Other				
1,707,735	M	5	Het	c.3742C > T	p.Arg1248Trp	Motor	Congenital	Severe	Flat	HA(Bi)
				c.10251_10253delCTT	p.Phe3420del	FERM 2				
1,707,757	F	6	Het	c.5692C > T	P.Arg1898*	Motor	Congenital	Severe	Flat	HA(Bi)
				c.7396-1G > A	Splice site	Other				
1,707,773	F	3	Het	c.4252G > A	p.Gly1418Arg	Motor	Congenital	Profound	Flat	CI(R)
				c.4596 + 1G > A	Splice site	Motor				
1,897,966	M	6	Het	c.5964 + 3G > A	Splice site	Other	Congenital	Profound	Flat	CI(R)
				c.8791del	p.Trp2931Glyfs*103	SH3				
1,897,999	F	28	Het	c.4747 T > C	p.Ser1583Pro	Motor	Congenital	Profound	Flat	Nothing
				c.10502 T > A	p.Leu3501Glu	Other				
1,801,948	M	16	Het	c.4597-2A > G	Splice site	Motor	Congenital	Profound	Flat	HA(Bi)
				c.8077del	p.Leu2693Cysfs*45	FERM 1				
				c.10420A > G	p.Ser3474Gly^a^	FERM 2				

Abbreviations: *L* Left, *R* Right, *Bi* Bilateral, *CI* Cochlear Implant, *HA* Hearing aid, *F* Female, *M* Male

^a^ The pathogenicity of the Ser3474Gly variant is quite problematic due to the finding of two homozygotes with an unknown phenotype in the ExAC database, and because it has been reported as not pathogenic in a Korean *DFNB3* family

### Clinical findings

**Fig. 1:**
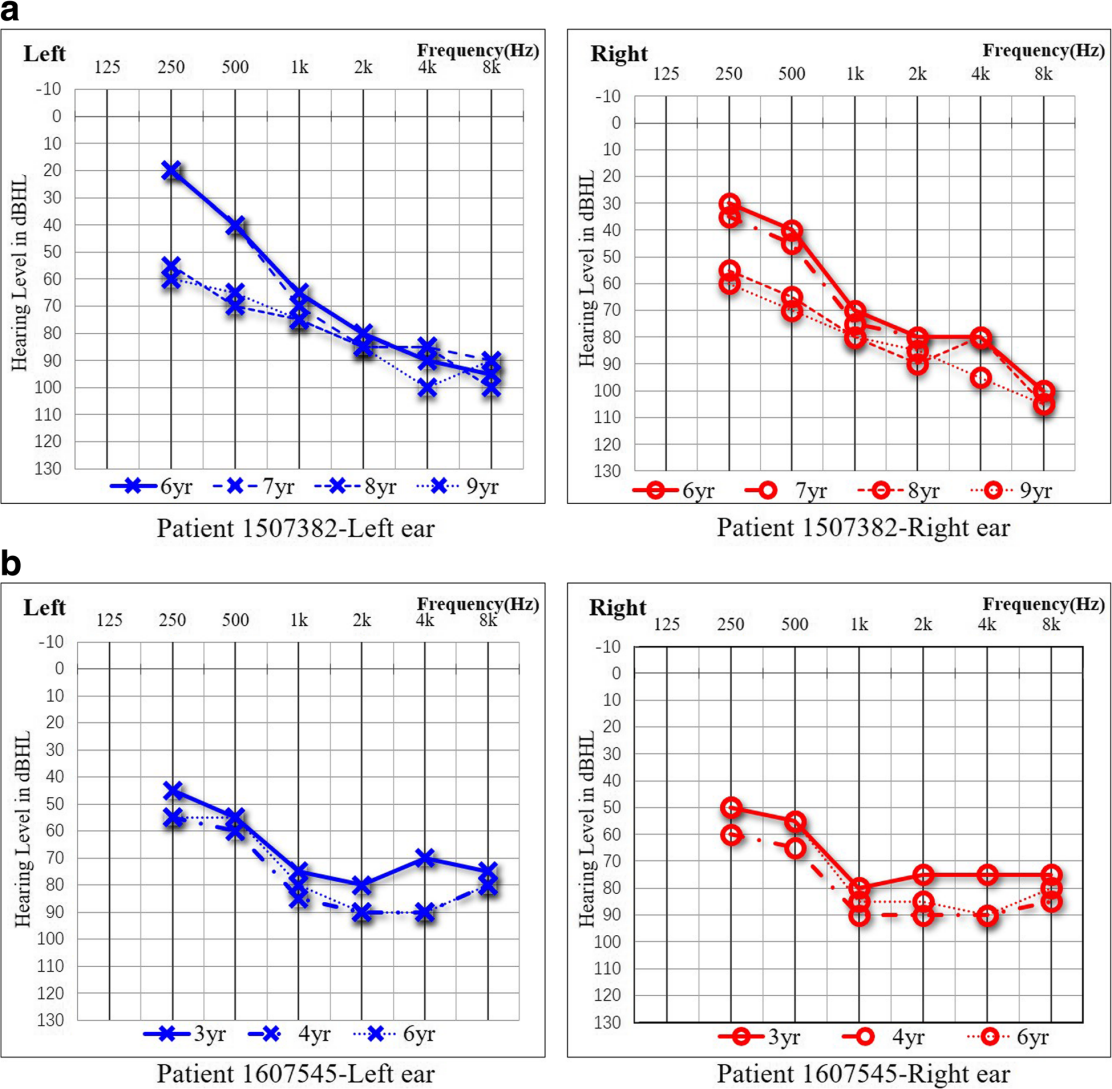
Audiograms of case 1,507,382 and 1,607,545. **a** The solid line represents the auditory thresholds measured when case 1,507,382 was 6 years old. The audiogram shows bilateral symmetrical severe sensorineural HL mainly affecting high frequencies, with residual hearing at low frequencies. Dashed lines indicate the results of auditory follow-up at seven, eight and nine years old, respectively. The pure tone threshold of this child increased by approximately 30–40 dB HL at 0.25 kHz–0.5 kHz and 10–20 dB HL at 1 kHz–8 kHz in the bilateral ears. **b** The solid line represents the auditory thresholds measured when case 1,607,545 was three years old, and the dashed lines display auditory thresholds when the child was four and six years old, respectively. The audiograms of the case 1,607,545 show bilateral residual hearing at low frequencies with progressive hearing loss at all frequencies. HL, hearing loss; dB, decibels; Hz, Hertz

Among the 15 individuals with *MYO15A* variants, patients 1,607,486–1 and 1,607,486–2 are siblings from the same family, while the remaining 13 individuals are from different families. The details of the clinical findings, including gender, age of test, age of onset, and hearing impairment phenotype, are summarized in Table 1. We further analysed the auditory phenotype in these patients with *MYO15A* gene variants. We found that there were at least two distinct auditory phenotypes that could be classified as a relatively severe and a less severe phenotype, based on diverse hearing impairment characteristics. The majority of these 15 cases presented with a severe auditory phenotype including bilateral, symmetrical and congenital severe or profound hearing loss at all frequencies; however, patients 1,507,382 and 1,607,545 presented with a milder phenotype. Patient 1,507,382, a 9-year-old boy, was first diagnosed with sensorineural hearing loss at 5 years of age. However, this boy’s enunciation was unclear; therefore, we speculated that this boy had pre-lingual hearing loss. Audiograms for this boy displayed bilateral, symmetrical severe sensorineural HL mainly affecting high frequencies, with residual hearing at low frequencies. Furthermore, we annually performed follow-up auditory assessments for this boy from 6 to 9 years of age. The result showed that pure tone thresholds had increased to approximately 30–40 dB HL at 0.25 kHz–0.5 kHz and 10–20 dB HL at 1 kHz–8 kHz in the bilateral ears (Fig. 1a). Patient 1,607,545 is another case with a milder phenotype who was found with congenital hearing impairment through newborn hearing screening using otoacoustic emission (OAE). As for the previous patient, we performed three follow-up assessments for this girl at the ages of 3, 4 and 6 years, using visual reinforcement audiometry or play audiometry. Audiograms showed bilateral progressive severe to profound sensorineural HL, mainly affecting high frequencies, with moderate residual hearing at low frequencies (Fig. 1b).

### Literature review of the genotype-phenotype correlation of *MYO15A* variants

**Fig. 2:**
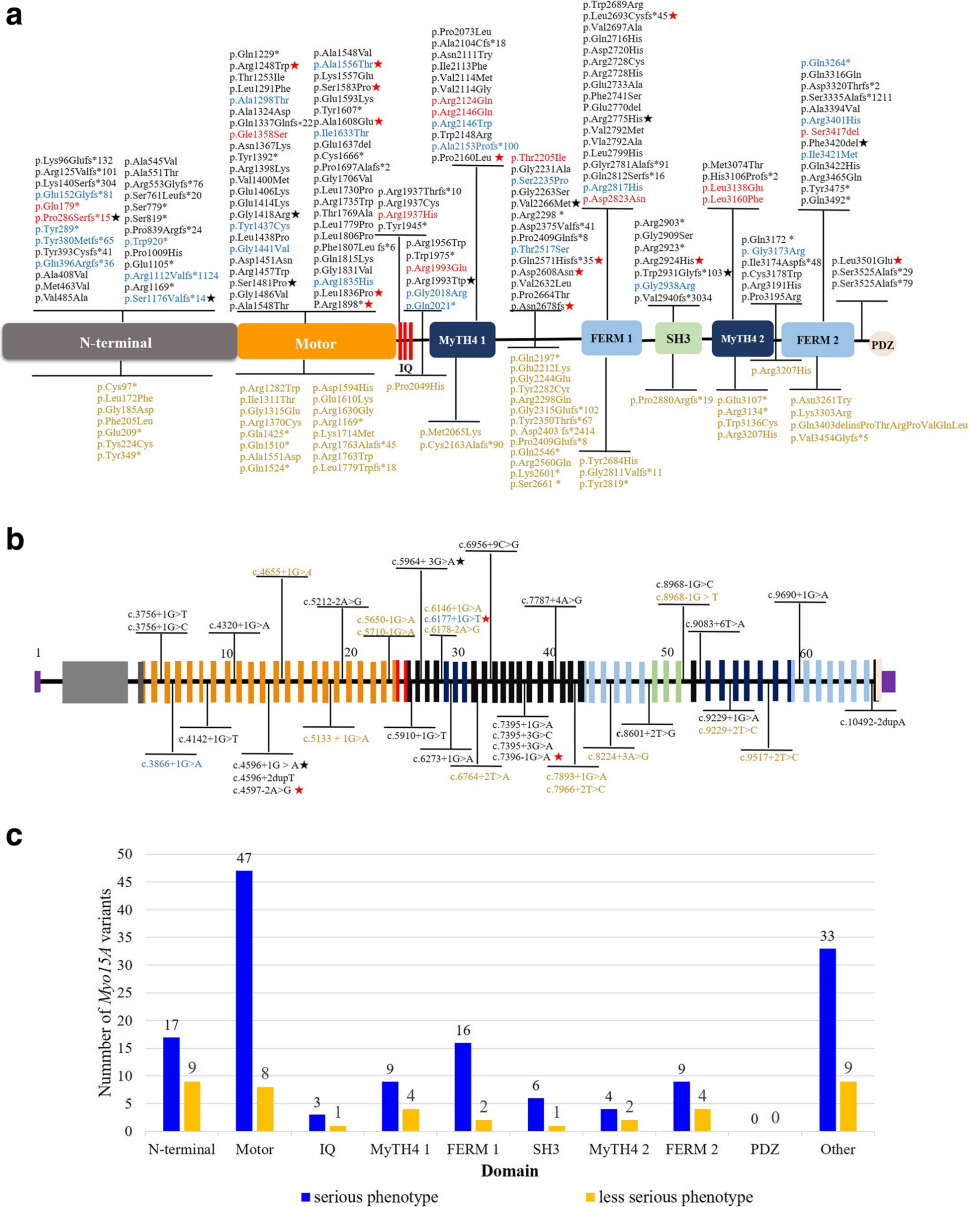
Overview of known *MYO15A* variants and related hearing phenotypes. The blue words indicate variants associated with milder auditory phenotypes, the red words indicate variants associated with reported both severe and milder phenotypes. In addition, variants without reported hearing loss phenotypes are expressed in brown words. Sixteen red stars indicate novel variants of *MYO15A* in this study, and eleven black stars show previously reported variants of *MYO15A* in this study. **a** Except for splice site variants of *MYO15A,* 159 *MYO15A* variants with reported hearing phenotypes were noted above the schematic diagram, 52 variants without reported hearing phenotypes were listed under it. **b** Thirty-eight splice site variants of *MYO15A* were indicated on the border between the exons flanking and affected introns. The pathogenicity of p.Ser3474Gly is ambiguous, so it is not listed here. **c** The number of identified *MYO15A* variants with two different auditory phenotypes in every affected domain is displayed

**Fig. 3:**
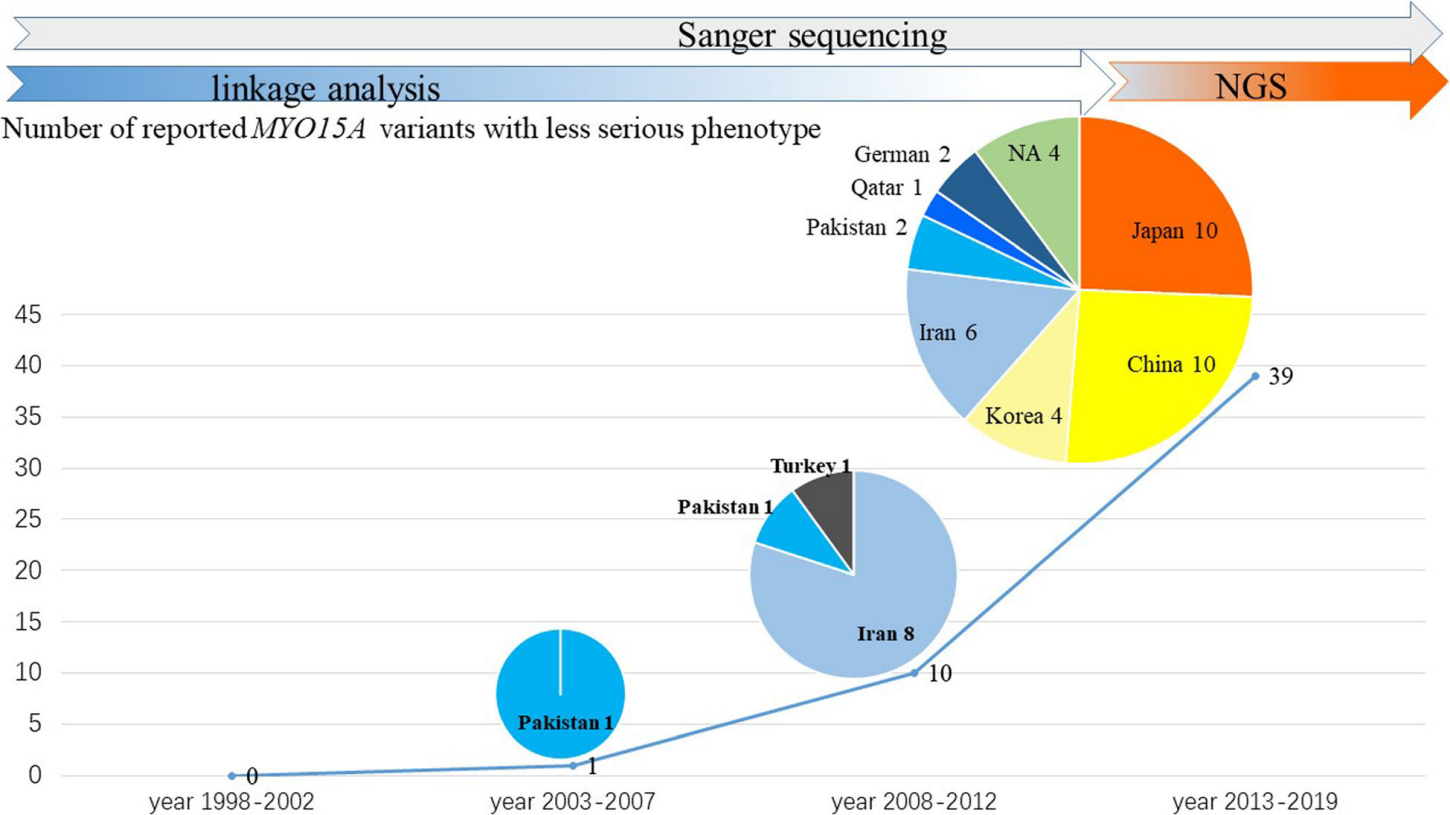
The number of previously reported *MYO15A* variants with a milder auditory phenotype in four periods (years 1998–2002, 2003–2007, 2008–2012 and 2013–2019). Several auditory phenotypes are considered as the milder auditory phenotype, such as mild /moderate /moderate-to-severe hearing loss, progressive post-lingual deafness and severe hearing loss with a typical slope toward high frequencies (residual hearing at low frequencies). The meaning of the numbers after the name of each country is the number of reported *MYO15A* variants with a milder auditory phenotype of each country in different periods. Following the rapid advance of molecular genetic techniques, there was an increasing number of reported *MYO15A* variants with milder auditory phenotypes in more countries worldwide, especially in Southeast Asia, such as China, Japan, and Korea. NA mean ethnic origin of patients were unavailable

Finally, a total of 249 *MYO15A* variants associated with *DFNB3* were reported in the literature, including 38 splice site variants (Fig. 2a and b) [1–3, 5, 9, 10, 12–69]. After filtering out the 65 variants without reported hearing loss phenotypes, we endeavoured to explore the relationship between the genotypes and auditory phenotypes in the remaining 184 variants of *MYO15A*. To our surprise, almost all domains had been reported to be correlated with a milder phenotype; however, variants encoding the N-terminal domain were more likely to cause a milder phenotype (Fig. 2c). Since 2007, when the first *MYO15A* variant case with a milder hearing impairment phenotype from Pakistan was published, advances in high-throughput techniques have led to a fast-growing number of reported *MYO15A* variants with a milder auditory phenotype in additional countries, especially in Southeast Asia (Fig. 3). Table 2 shows details of all reported *MYO15A* variant cases associated with milder phenotypes.

**Table 2:** Overview of all known cases of *MYO15A* variations with milder hearing loss phenotype

Ethnic Origin	References	Zygosity	cDNA (NM_016239.3)	Protein change (NP_057323.3)	Variant type	Exon/intron^a^	Protein domain	Hearing impairment phenotype		
								Age of onset(year)	Severity (PTA)	Type of audiometry
China	This study	Het	c.4666G > A	p.Ala1556Thr	missense	15	Motor	Pretlingual(5) Progressive	Severe /residual hearing of low frequencies	down-sloping
			c.6177 + 1G > T	Splice site	splicing	*28*	MyTH4 1			
China	This study	Het	c.855dupT	p.Pro286Serfs*15	frameshift	2	N-terminal	Congenital	Severe /residual hearing of low frequencies	down-sloping
			c.3524dupA	p.Ser1176Valfs*14	frameshift	2	N-terminal			
			c.7822G > A	p.Asp2608Asn	missense	41	Other			
Iran	Mehregan,2018	Hom	c.6436C > T	p.Arg2146Trp	missense	30	MyTH4 1	Pre-lingual	Moderate to severe	down-sloping
Korea	Chang, 2018	Het	c.5504G > A	p.Arg1835His	missense	22	Motor	Post-lingual, Progressive	Severe /residual hearing of low frequencies	down-sloping
			c.10245_10247delCTC	p.Ser3417del	missense	64	FERM 2			
Korea	Chang, 2018	Het	c.9790C > T	p.Gln3264*	nonsense	60	FERM 2	Post-lingual, progressive	Severe /residual hearing of low frequencies	down-sloping
			c.10263C > G	p.Ile3421Met	missense	64	FERM 2			
Pakistan	Naz,2017	Hom	c.3866 + 1G > A	Splice site	splicing	*5*	Motor	NA	Moderate	NA
Pakistan	Naz,2017	Hom	c.8158G > A	p.Asp2720His	missense	45	FERM 1	NA	Moderate	NA
China	Li, 2016	Hom	c.3524dupA	p.Ser1176Valfs*14	frameshift	2	N-terminal	Congenital	Severe/ residual hearing of low frequencies	down-sloping
NA	Sloan,2016	Het	c.7550C > G	p.Thr2517Ser	missense	39	Other	Congenital	Mild moderate asymmetric	NA
			c.8812G > A	p.Gly2938Arg	missense	51	SH3			
NA	Sloan,2016	Het	c.4310A > G	p.Tyr1437Cys	missense	11	Motor	Postlingual Childhood	Mild moderate	NA
			c.10202G > A	p.Arg3401His	missense	63	FERM 2			
Iran	Sloan,2015	NA	c.2759G > A	p.Trp920*	nonsense	2	N-terminal	Congenital	Moderate	NA
Iran	Sloan,2015	NA	c.4907_4909delAGG	p.Glu1637del	indel	17	Motor	Post-lingual	Severe to profound	NA
Iran	Sloan,2015	NA	c.5810G > A	p.Arg1937His	missense	24	IQ	Post-lingual	Severe	NA
Iran	Sloan,2015	NA	c.6437G > A	p.Arg2146Gln	missense	30	MyTH4 1	Postlingual	Severe	NA
Iran	Sloan,2015	NA	c.8467G > A	p.Asp2823Asn	missense	48	FERM1	Congenital	Moderate	NA
China	Gu,2015	Het	c.4322G > T	p.Gly1441Val	missense	12	Motor	Congenital	Severe / residual hearing of low frequencies	down-sloping
			c.4898 T > C	p.Ile1633Thr	missense	17	Motor			
China	Gu,2015	Het	c.3892G > A	p.Ala1298Thr	missense	6	Motor	Congenital	Severe/ residual hearing of low frequencies	down-sloping
			c.8450G > A	p.Arg2817His	missense	47	FERM1			
Japan	Miyagawa,2015	Het	c.535G > T	p.Glu179*	nonsense	2	N-terminal	Congenital	Moderate	down-sloping
			c. 9413 T > A	p.Leu3138Gln	missense	57	MyTH4 2			
Japan	Miyagawa,2015	Het	c.5978G > A	p.Arg1993Glu	missense	27	Other	Postlingual(8)	Severe/ residual l hearing of low frequencie	down-sloping
			c. 9517G > A	p. Gly3173Arg	missense	57	Other			
Japan	Miyagawa,2015	Het	c.5978G > A	p.Arg1993Glu	missense	27	Other	Postlingual(10)	Severe/ residual l hearing of low frequencie	down-sloping
			c.6487delG	p.Ala2153Profs*100	frameshift	30	MyTH4 1			
Japan	Miyagawa,2015	Het	c.6703 T > C	p.Ser2235Pro	missense	32	Other	Postlingual(12)	Moderate/ residual l hearing of low frequencies	down-sloping
			c.10263C > G	p.Ile3421Met	missense	64	FERM 2			
Japan	Miyagawa,2015	Het	c. 9413 T > A	p.Leu3138Gln	missense	57	MyTH4 2	Congenital	Profound / residual hearing of low frequencies	down-sloping
			c.9478C > T	p. Leu3160Phe	missense	57	MyTH4 2			
Qatar	Vozzi, 2014	Hom	c.453_455delCGAinsTGGACGCCTGGTCGGGCAGTGG	p.Glu152Glyfs*81	frameshift	2	N-terminal	Progressive	Profound	flat
German	Vona,2014	Het	c.1137delC	p.Tyr380Metfs*65	frameshift	2	N-terminal	Pre-lingual(3) Progressive	Normal between 0.125 and 0.25 kHz/ steeply sloping to severe HL	down-sloping
			c.7124_7127delACAG	p.Asp2375Valfs*29	frameshift	35	other			
Iran	Fattahi,2012	Hom	c.5305A > G	p.Thr1769Ala	missense	20	Motor	Congenital	Severe to profound /residual hearing of low frequencies	down-sloping
Iran	Fattahi,2012	Hom	c.5925G > A	p.Trp1975*	nonsense	26	Other	Congenital	Severe to profound/residual hearing of low frequencies	down-sloping
Iran	Fattahi,2012	Het	c.5419_-_21delT	p.Phe1807Leu fs*6	frameshift	22	Motor	Congenital	Severe to profound/residual hearing of low frequencies	down-sloping
			c.1387A > G;	p.Met463Val	missense	2	N-terminal			
Iran	Fattahi,2012	Hom	c.8467G > A	p.Asp2823Asn	missense	48	FERM1	Congenital	Severe to profound/residual hearing of low frequencies	down-sloping
Iran	Fattahi,2012	Hom	c.5810G > A	p.Arg1937His	missense	24	IQ	Congenital	Severe to profound/residual hearing of low frequencies	down-sloping
Iran	Fattahi,2012	Hom	c.4904_-_4907delGAG	p.Glu1637del	indel	17	Motor	Congenital	Severe to profound/residual hearing of low frequencies	down-sloping
Pakistan	Bashir,2012	Hom	c.1185dupC	p. Glu396Argfs*36	frameshift	2	N-terminal	Congenital	Moderate to severe /residual hearing of low vfrequencies	down-sloping
Turkey	Cengiz,2010	Hom	c.867C > G	p.Tyr289*	nonsense	2	N-terminal	Progressive	Moderately severe/residual hearing of low frequencies	down-sloping
Iran	Shearer,2009	Hom	c.6371G > A	p. Arg2124Gln	missense	30	MyTH4 1	NA	Severe /residual hearing of low frequencies	down-sloping
Pakistan	Nal,2007	Hom	c.3334delG	p.Arg1112Valfs*1124	frameshift	2	N-terminal	Congenital	Severe /residual hearing of low frequencies	down-sloping

^a^ Location of *MYO15A* splicing variants in introns are shown in italic letters. *Abbreviations*: *NA* not available, *Hom* homozygous variant, *Het* compound heterozygous variant

**Fig. 4:**
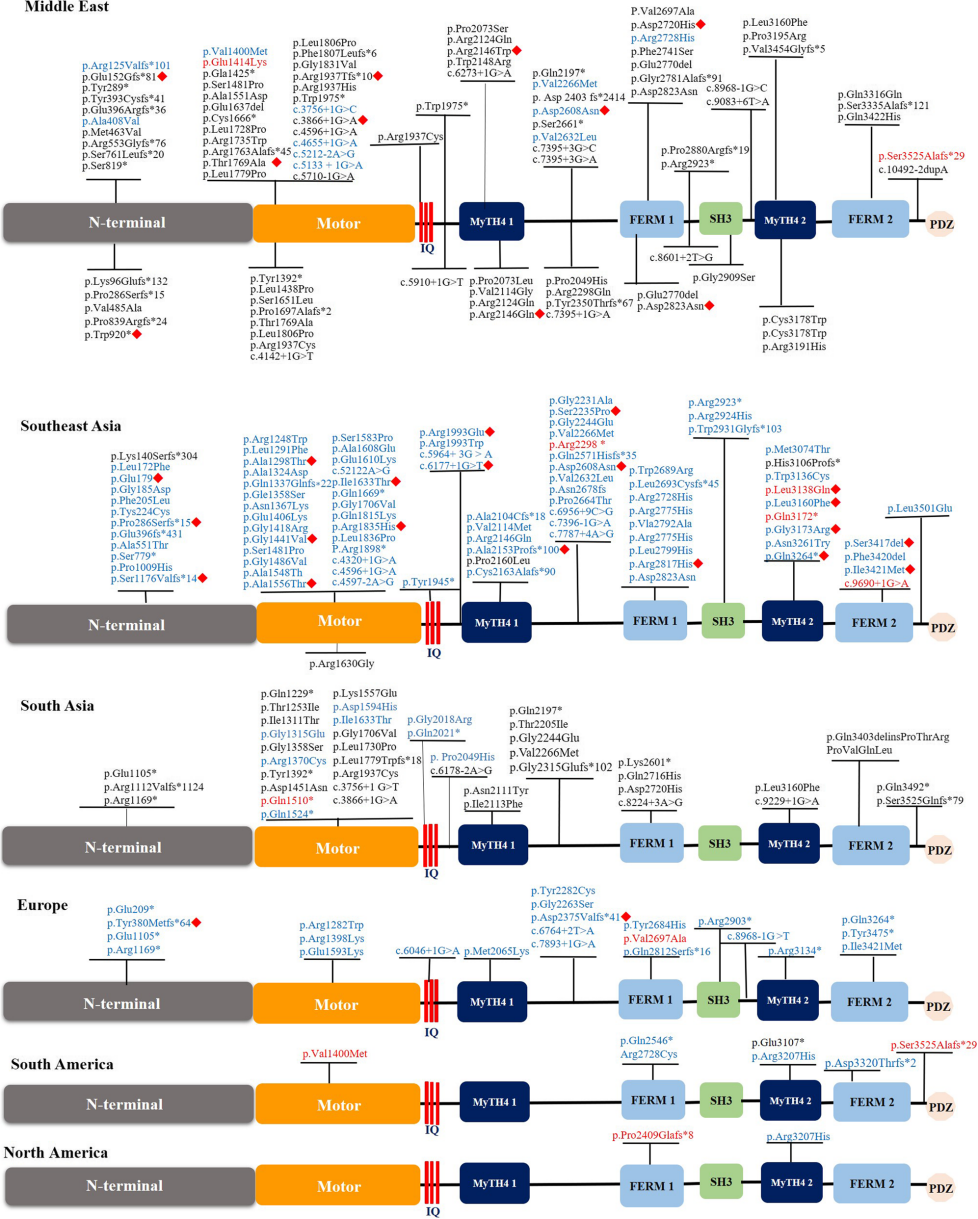
The spectrum of *MYO15A* variants in different populations. The black words indicate homozygous variants, the blue words indicate compound heterozygous variants and the red words represent *MYO15A* variants that were reported with both compound heterozygous and homozygous variants. In all populations, *MYO15A* variants with known zygosity types were noted above the schematic diagram, and variants with unknown zygosity types were noted below it. Red diamond-shaped signs in all populations designate *MYO15A* variants with milder auditory phenotypes

**Fig. 5:**
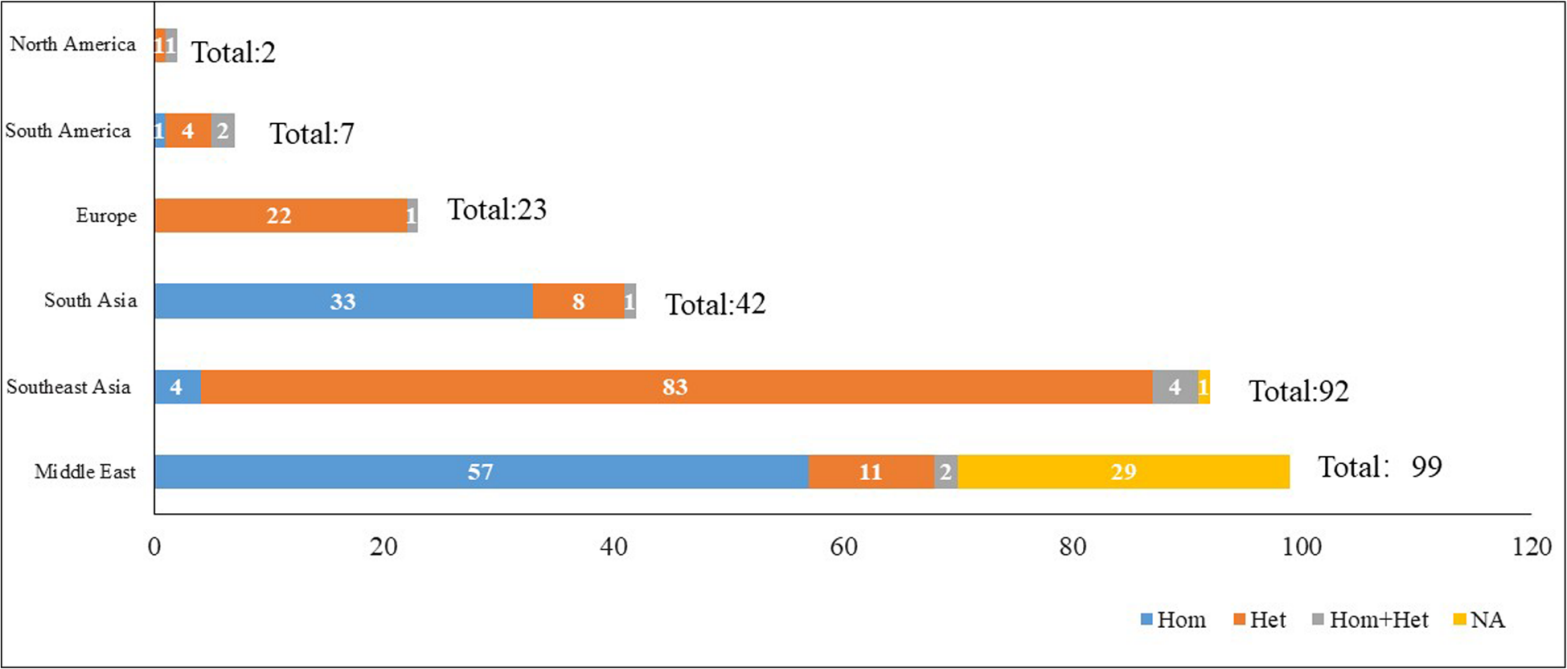
The number of identified variants in different populations. The rank (from high to low) of reported number of *MYO15A* variants in different populations in the Middle East, Southeast Asia, South Asia, Europe, South America, and North America. This illustrates that in the Middle East and South Asia, most *MYO15A* variants were homozygous variants. On the other hand, more compound heterozygous variants were identified in Southeast Asia and Europe

We further summarized the genotype-phenotype correlation of the *MYO15A* variant in different populations (Fig. 4). In our review, the reported number of *MYO15A* variants in individuals from Middle East, Southeast Asia, South Asia, Europe, South America and North America was 99, 92, 42, 23, 7 and 2, respectively, and the reported number of *MYO15A* variants from Middle Eastern was largest. However, the number of known *MYO15A* variants in Southeast Asia has increased in recent years. Moreover, based on our review, we found a notable genetic characteristic that occurs in different populations. In the Middle East and South Asia, most *MYO15A* variants were homozygous variants, probably resulting from the custom of consanguineous marriage in these areas. However, more compound heterozygous variants have been identified in Southeast Asia and Europe in recent years (Fig. 5).

## Discussion

In 1995, the *DFNB3* locus was first mapped to chromosome 17p-17q12 by linkage analysis in two large multi-generational non-consanguineous families from a remote Indonesian village, Bengkala, where 2.2% of the population (47/2185) had severe-to-profound hearing loss. The locus was subsequently further refined to chromosome 17p11.2 [70, 71]. Since 1998, when three homozygous variants of *MYO15A* were initially identified in three multigenerational ARNSHL families from Indonesia and India [5], more than 200 variants have been reported to date. Our results expanded the spectrum of sequence variants in the *MYO15A* gene. In the present study, we identified 16 novel possible pathogenic variants, including one nonsense variant, two ±1 or 2 splice site variants, three frameshift variants with evidence of pathogenicity and 9 missense variants (p.Arg1248Trp, Ala1556Thr, p.Ser1583Pro, p.Ala1608Glu, p.Leu1836Pro, p.Pro2160Leu, p.Asp2608Asn, Arg2924His, p.Leu3501Glu).

The *MYO15A* gene contains 67 exons and encodes several alternatively spliced transcripts, and the longest mRNA transcript contains 3530 amino acids. It includes a long N-terminal extension encoded by giant exon 2, an ATPase motor domain, two light chain binding IQ motifs, and a tail region containing two myosin-tail homology 4 (MyTH4) domains, two band 4.1 superfamily (ezrin, radixin and moesin) (FERM), a Src-homology-3 (SH3) domain and a C terminal class I PDZ-ligand domain. Additionally, based on the presence or lack of giant exon 2, there are two alternatively spliced transcripts, isoform classes II and I. Myosin XVa is a member of the unconventional myosin superfamily, which is indispensable in the graded elongation of stereocilia of cochlear and vestibular hair cells. Whirlin is a scaffolding protein that is essential for maintaining normal human hearing (DFNB31, OMIM #607084) and sight (USH2D, OMIM #611383). EPS8 is an actin capping protein and is similarly essential for human hearing (DFNB102, OMIM #615974). Myosin XVa can transport whirlin and Eps8 to the tip of stereocilia and interacts with them to form a stereocilia tip complex, which can facilitate the extremely important transformation of microvilli into mature stereocilia [72]. *Shaker-2* (*Myo15sh2*) mice exhibit profound hearing loss and abnormal vestibular function caused by short stereocilia and by losing the normal staircase structure of stereocilia in hair cells [7, 8].

In our literature review, the number of *MYO15A* variants affecting the motor domain is the largest of all domains (Fig. 2c). The motor domain consists of ATP- and actin-binding sites, which can generate force and move actin filaments; therefore, it is not surprising that motor domain dysfunction affected by the *MYO15A* variant will lead to shorter stereocilia with an ectopic staircase structure of stereocilia associated with a severe deafness phenotype [73]. Earlier studies showed that the motor and tail regions of myosin XVa were critical for normal auditory structure and function, whereas the exact biological function of the large N-terminal extension initially remained obscure [73]. However, recent study demonstrated that the 133-kDa N-terminal domain enables myosin 15 to maintain mechanotransducing stereocilia and is essential for hearing [74]. In 2007, two mutant alleles encoding the N-terminal domain were identified that provided evidence that the class I isoform of myosin XVa with an N-terminal extension is essential for normal hearing in humans [22]. More particularly, compared with the previous phenotype involving profound hearing loss at all frequencies, a milder phenotype with residual hearing at low frequencies (250 Hz, 500 Hz) was shown for the first time. In addition, abnormally short stereocilia could be restored to a wild type-like staircase architecture by simply transfecting myosin XVa Isoform II in shaker-2 mice. Therefore, it was hypothesized that a variant in the N-terminal extension domain might be associated with functional problems or minor structural defects in the staircase architecture of hair cell stereocilia [22]. Further, the results from the later mouse model experiments suggested that the pathogenic mechanisms of the two isoforms were different. Isoform II with no N-terminal domain can transport whirlin and EPS8 to the tips of hair cell stereocilia and drive elongation of the core actin cytoskeleton. Alternatively, Isoform I, with an N-terminal domain, is responsible for stabilizing the actin cytoskeleton and preventing the disassembly of mature mechano-transduction sites [2] . This finding suggests that there may be a genotype-phenotype correlation. Variants in giant exon 2, which may seldom result in drastic changes in the protein structure and function of the inner ear, may be involved in a variety of milder hearing loss phenotypes, such as residual hearing at low frequencies, moderate-to-severe hearing loss and progressive post-lingual deafness. In our study, we reported one case (1607545) with two compound heterozygous variants (p.Pro286Serfs*15, p.Ser1176Valfs*14) in 2 exons encoding the N-terminal domain who showed residual hearing at low frequencies. It is rather remarkable that this homozygous *MYO15A* variant (p.Ser1176Valfs*14) had recently been identified in a non-consanguineous Chinese family. The audiograms of two affected siblings also showed moderate residual hearing at identical low frequencies, which seems to provide additional evidence of an association between the mutant allele p.Ser1176Valfs*14 and a less severe auditory phenotype, especially in Chinese families [13]. In addition, our literature review illustrated that variants in the N-terminal domain were more frequently related to less sever hearing impairment than variants in the other domains.

A genotype-phenotype correlation of *MYO15A* variants should predict hearing loss tendency according to affected domain, which will inform the choice of optimal hearing rehabilitation methods for individuals [43]. For instance, on the basis of this hypothetical genotype-phenotype correlation, cases with variants affecting the N-terminal extension seem to need cochlear implantation with low frequency hearing retention or hearing aids due to their milder hearing loss. However, upon review of the relevant literature, we found that the genotype-phenotype correlation of *MYO15A* was much more complicated. First, recent studies showed that some pathologic variants affecting domains (motor, IQ, MyTH4 1, FERM 1, SH3, MyTH4 2, FERM 2) that would have been expected to cause a severe hearing phenotype, actually resulted in a milder auditory phenotype. The above relationship was further confirmed by our finding that case (1507382) showed residual low-frequency hearing and compound heterozygosity for *MYO15A* (c.4666G > A, c.6177 + 1G > T), affecting the motor and MyTH4 1 domains. Conversely, some variants associated with dysfunction of the N-terminal domain cause congenital severe to profound hearing loss instead of a milder phenotype. In addition, we reviewed 12 *MYO15A* variants associated with both severe and milder phenotypes (Table 3). We found that affected members of the same family expressed different auditory phenotypes, even though they carried the same *MYO15A* variants [17, 60]. For instance, Cengiz et al. [17] reported a Turkey ARNSHL family carried a homozygous *MYO15A* variant (p.Tyr289*) affecting the N-terminal domain. The audiograms of three affected siblings revealed congenital severe to profound sensorineural hearing loss at all frequencies, while their mother showed progressive severe sensorineural hearing loss of a milder form, which suggested the presence of a modifier. To our knowledge, in general, autosomal dominant non-syndromic hearing loss (ADNSHL) were associated with post-lingual hearing impairment, whereas ARNSHL were associated with congenital or pre-lingual hearing impairment. However, according to the results of our literature review, there were 8 *MYO15A* variant cases from Japan and Korea with unconventional post-lingual hearing impairment (Table 2). It should be noted that unlike the majority of *MYO15A* variant cases with a stable auditory phenotype, progressive hearing loss, especially at low frequencies, was observed in both affected individuals with a less severe auditory phenotype in our study based on 3-year auditory follow-ups, which further demonstrated the phenotypic diversity of the *MYO15A* variant.

**Table 3:** Reported variants of *MYO15A* related to both a severe hearing loss phenotype and a milder hearing loss phenotype

cDNA (NM_016239.3)	Protein chang (NP_057323d.3)	Exon/Intron	Proteindomain	Zygosity	Hearing impairment phenotype		Ethnic Origin	References
					Age of onset(year)	Severity		
c.535G > T	p.Glu179*	2	N-terminal	Het	Congenital	Profound	Korea	ParK,2014
				Het	Congenital	Moderate	Japan	Miyagawa,2015
c.855dupT	p.Pro286Serfs*15	2	N-terminal	Het	Pre-lingual progressive	Severe /Residual low frequencies hearing	China	this study
				Hom	Congenital	Severe to profound	Iran	Sloan,2015
c.4072G > A	p.Gle1358Ser	9	Motor	Het	Post-lingual	Mild	Japan	Miyagawa,2015
				Hom	Congenital	Profound	Pakistan/India	Friedman,2002
c.5810G > A	p.Arg1937His	24	IQ	NA	Post-lingual	Severe	Iran	Sloan,2015
				Het	Childhood	Severe to profound	Iran	Sloan,2016
				Hom	Congenital	Severe to profound	Iran	Fattahi,2012
c.5978C > T	p.Arg1993Gln	27	Other	Het	Post-lingual (8)	Severe	Japan	Miyagawa,2015
				Het	Post-lingual (10)	Severe	Japan	Miyagawa,2015
				Het	Pre-lingual	Severe	Japan	Miyagawa,2013
c.6371G > A	p.Arg2124Gln	30	MyTH4 1	NA	Congenital	Severe to profound	Iran	Sloan,2016
				Hom	NA	Severe to profound,	Iran	Shearer,2009
				Hom	NA	Residual low frequencies hearing	Iran	Shearer,2009
c.6437G > A	p.Arg2146Gln	30	MyTH4 1	NA	Post-lingual	Severe	Iran	Sloan,2015
				Het	Congenital	Severe to profound	Korea	Woo,2013
c.6614C > T	p.Thr2205Ile	31	Other	Het	Congenital	Severe to profound	Iran	Sloan,2016
				Hom	Congenital	Severe to profound	Pakistan	Nal,2007
c.8467G > A	p.Asp2823Asn	48	FERM 1	NA	Congenital	Moderate	Iran	Sloan,2015
				Homo	Congenital	Profound	Israel	Brownstein,2014
				Homo	Congenital	Severe to profound	Iran	Fattahi,2012
c. 9413 T > A	p.Leu3138Gln	57	MyTH4 2	Het	Congenital	Moderate	Japan	Miyagawa,2015
				Homo	Congenital	Profound	Japan	Miyagawa,2015
				Het	Congenital	Profound/residual low frequencies hearing	Japan	Miyagawa,2015
c.9478C > T	p.Leu3160Phe	57	MyTH4 2	Het	Childhood	Profound	Iran	Sloan,2016
				Het	Congenital	Profound	Japan	Miyagawa,2015
				Het	Congenital	Profound/ residual low frequencies hearing	Japan	Miyagawa,2015
				Het	Congenital progressive	Severe to profound	Japan	Miyagawa,2013
				Het	Pre-lingual	Severe	Japan	Miyagawa,2013
				Het	Congenital	Severe to profound	Japan	Miyagawa,2013
c.10249_10251delTCC	p. Ser3417del	64	FERM 2	Het	Congenital	profound	Japan	Miyagawa,2015
				Het	Post-lingual progressive	Residual low frequencies hearing	Korea	Chang,2018

*Abbreviations*: *NA* not available, *Hom* homozygous variant, *Het* compound heterozygous mutation variant

Genetic modifiers or environmental factors might contribute to the complex genotype-phenotype correlations of *MYO15A* variants [9]. In addition, it is noteworthy that in contrast to conventional genetic tests such as linkage analysis, the more cost-effective and highly efficient technology of next-generation sequencing (NGS), which was commonly employed after 2012, saw a dramatic increase in the number of pathogenic variants identified. With the use of NGS, more sporadic cases with milder auditory phenotypes from non-consanguineous families have the opportunity to be involved in genetic studies to identify pathogenic gene variants. Therefore, more *MYO15A* compound heterozygous variants have been identified from various ethnic groups, without being limited to consanguineous families of the Middle East. This enriches the pool of known genotypes and phenotypes of *MYO15A* variants. It is also worth noting that there were several cases of progressive hearing loss both in recent reports and in our present study, therefore it is necessary to focus not only on age of onset but also age of auditory testing when performing genotype-phenotype correlation analyses. Gathering sufficient historical data and long-term follow-up related to auditory testing are fundamental to establishing specific genotype-phenotype correlations.

## Conclusions

In summary, we identified 16 novel variants and 12 reported pathogenic *MYO15A* variants in 15 NSHL patients. Additionally, we described two distinct auditory phenotypes, a severe phenotype and a milder phenotype, in the present study. Our work extends the spectrum of *MYO15A* pathogenic variants, enriches our knowledge of auditory phenotypes, and deepens our understanding of the *MYO15A* genotype-phenotype correlation. In the future, additional functional studies including more cases with phenotypes based on accurate assessments of hearing loss phenotypes are necessary to ascertain the *MYO15A* genotype-phenotype correlation and provide optimal rehabilitation methods for affected individuals through precisely targeted genetic counselling.

## Additional files

Additional file 1: **Table S1.** List of 127 targeted genes or related regions. (DOCX 15 kb)

Additional file 2: **Figure S1.** Pedigrees of the families carried *MYO15A* variants. (PDF 420 kb)

Additional file 3: **Table S2.** Variants of *MYO15A* detected in this study. (DOCX 25 kb)

## Abbreviations

ABR: Auditory brainstem response
ADNSHL: Autosomal dominant non-syndromic hearing loss
ARNSHL: Autosomal recessive non-syndromic hearing loss
ASSR: Auditory steady state response
BWA: Burrows-Wheeler Aligner
DPOAE: Distortion product otoacoustic emission
GATK: Genome Analysis Toolkit
NGS: Next-generation sequencing
NSHL: Non-syndromic hearing loss
OAE: Otoacoustic emission
PTA: pure-tone audiometry
