# Supplementary material for: Genotype-phenotype correlation analysis of MYO15A variants in autosomal recessive non-syndromic hearing loss
Source: BMC Med Genet. 2019 Apr 5;20:60. doi: 10.1186/s12881-019-0790-2 (PMC6451310; doi:10.1186/s12881-019-0790-2)
Supplement: Supplementary file 1 — Table S1. List of 127 targeted genes or related regions. (DOCX 15 kb) [file 12881_2019_790_MOESM1_ESM.docx]

| Inheritance pattern | Genes |
| --- | --- |
| Autosomal recessive  nonsyndromic hearing  impairment | GJB2,GJB6,MYO7A,MYO15A,FOXI1,KCNJ10,SLC26A4,TMIE,TMC1,TMPRSS3,OTOF,CDH23,ATP2B2,GIPC3,STRC,OTOG,USH1C,TECTA,OTOA,PCDH15,RDX,GRXCR1,TRIOBP,CLDN14,MYO3A,WHRN,ESRRB,ESPN,MYO6,GJA1,HGF,ILDR1,MARVELD2,DFNB59,SLC26A5,LRTOMT,LHFPL5,BSND,MSRB3,LOXHD1,TPRN,GPSM2,PTPRQ,SERPINB6,GJB3 |
| X-link hereditary hearing  impairment | PRPS1,POU3F4,SMPX, |
| Autosomal dominant  nonsyndromic hearing  impairment | ACTG1,CCDC50,CEACAM16,COCH,CRYM,DFNA5,DIABLO,DIAPH1,DSPP,EYA4,GJB2,GJB3,GJB6,GRHL2,KCNQ4,MIR96,MYH14,MYH9,MYO1A,MYO6,MYO7A,POU4F3,SIX1,SLC17A8,TECTA,TJP2,TMC1,WFS1,DIAPH3 |
| Maternally inherited hearing impairment | MT-RNR1,MT-TS1, |
| Syndromic hearing  impairment | SERAC1,PDSS1,FGFR3,FGFR1,FGFR2,PHEX,DLX5,TNFRSF11B,COL2A1,COL11A1,COL9A1,COL9A2,COL4A3,COL4A4,COL4A5,BSND,SOX9,PAX2,GATA3,SLC19A2,IGF1,PAX3,MITF,SNAI2,EDNRB,EDN3,SOX10,HOXA1,SOBP,EYA1,SIX5,SIX1,CHD7,SEMA3E,SMAD4,FGF3,TCOF1,PRRX1,GLI3,HOXA2,  KCNQ1,KCNE1,CACNA1D,ALMS1,LRP2,TIMM8A,NDP,WFS1,OPA1,SLC4A11,MYO7A,USH1C,CDH23,PCDH15,USH1G,USH2A,ADGRV1,PDZD7,WHRN,CLRN1,MT-TK,MT-TE,MT-TL1,SLC26A4,KCNJ10,FOXI1 |

Supplementary Table 1. Targeted 127 genes or related regions list
