# Supplementary material for: Genotype-phenotype correlation analysis of MYO15A variants in autosomal recessive non-syndromic hearing loss
Source: BMC Med Genet. 2019 Apr 5;20:60. doi: 10.1186/s12881-019-0790-2 (PMC6451310; doi:10.1186/s12881-019-0790-2)
Supplement: Supplementary file 2 — Figure S1. Pedigrees of the families carried MYO15A variants. (PDF 420 kb) [file 12881_2019_790_MOESM2_ESM.pdf]

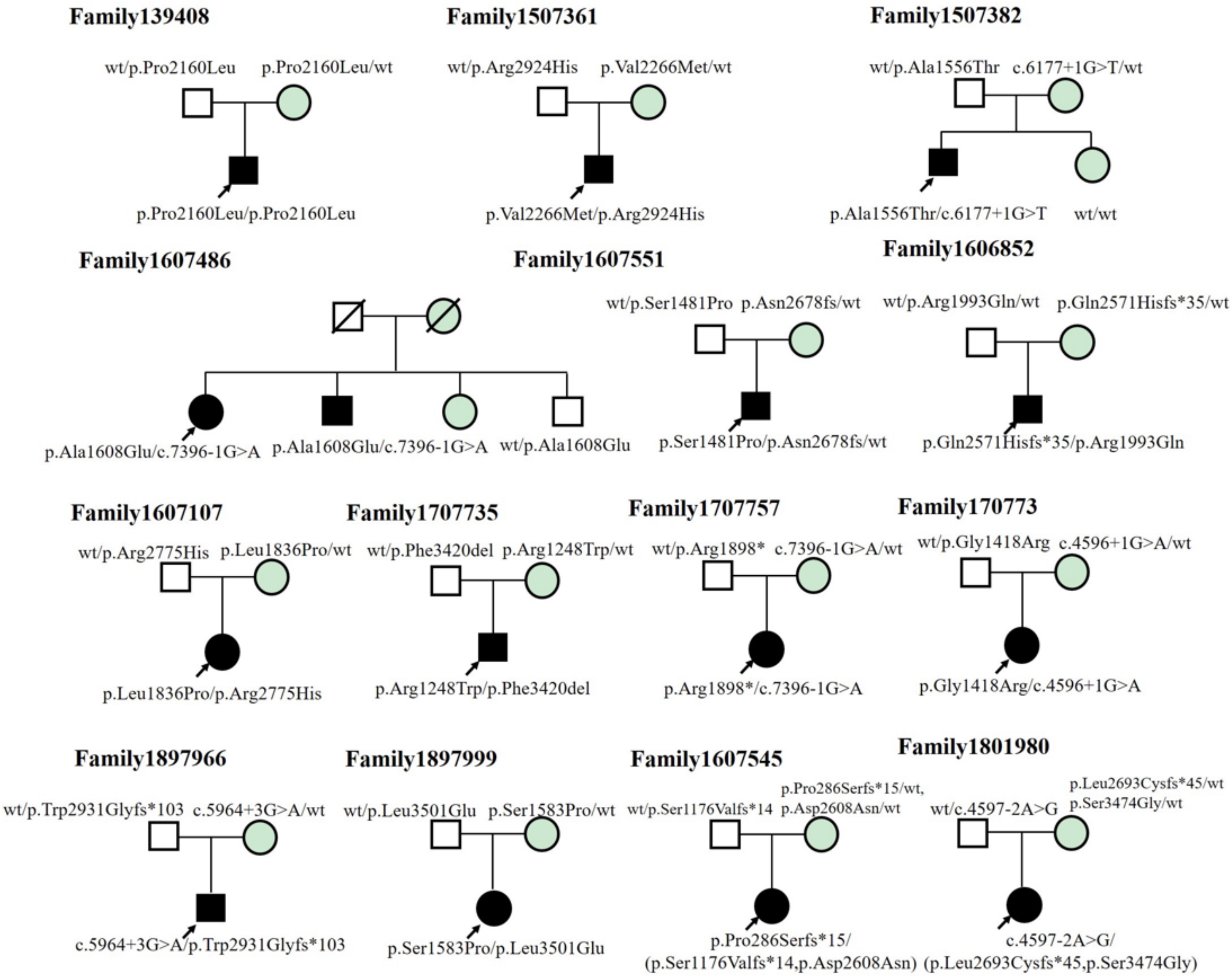
Family139408
wt/p.Pro2160Leu
p.Pro2160Leu/wt
p.Pro2160Leu/p.Pro2160Leu
Family1507361
wt/p.Arg2924His
p.Val2266Met/wt
p.Val2266Met/p.Arg2924His
Family1507382
wt/p.Ala1556Thr
c.6177+1G>T/wt
p.Ala1556Thr/c.6177+1G>T
wt/wt
Family1607486
p.Ala1608Glu/c.7396-1G>A
p.Ala1608Glu/c.7396-1G>A
wt/p.Ala1608Glu
Family1607551
wt/p.Ser1481Pro
p.Asn2678fs/wt
p.Ser1481Pro/p.Asn2678fs/wt
Family1606852
wt/p.Arg1993Gln/wt
p.Gln2571Hisfs*35/wt
p.Gln2571Hisfs*35/p.Arg1993Gln
Family1607107
wt/p.Arg2775His
p.Leu1836Pro/wt
p.Leu1836Pro/p.Arg2775His
Family1707735
wt/p.Phe3420del
p.Arg1248Trp/wt
p.Arg1248Trp/p.Phe3420del
Family1707757
wt/p.Arg1898*
c.7396-1G>A/wt
p.Arg1898*/c.7396-1G>A
Family170773
wt/p.Gly1418Arg
c.4596+1G>A/wt
p.Gly1418Arg/c.4596+1G>A
Family1897966
wt/p.Trp2931Glyfs*103
c.5964+3G>A/wt
c.5964+3G>A/p.Trp2931Glyfs*103
Family1897999
wt/p.Leu3501Glu
p.Ser1583Pro/wt
p.Ser1583Pro/p.Leu3501Glu
Family1607545
wt/p.Ser1176Valfs*14
p.Pro286Serfs*15/wt,
p.Asp2608Asn/wt
p.Pro286Serfs*15/
(p.Ser1176Valfs*14,p.Asp2608Asn)
Family1801980
wt/c.4597-2A>G
p.Leu2693Cysfs*45/wt
p.Ser3474Gly/wt
c.4597-2A>G/
(p.Leu2693Cysfs*45,p.Ser3474Gly)
