# Supplementary material for: Genotype-phenotype correlation analysis of MYO15A variants in autosomal recessive non-syndromic hearing loss
Source: BMC Med Genet. 2019 Apr 5;20:60. doi: 10.1186/s12881-019-0790-2 (PMC6451310; doi:10.1186/s12881-019-0790-2)
Supplement: Supplementary file 3 — Table S2. Variants of MYO15A detected in this study. (DOCX 25 kb) [file 12881_2019_790_MOESM3_ESM.docx]

**Supplement Table 2. Variants of *MYO15A* detected in This Study**

| **No.** | **Variant detected^a^** | | **Variation**  **type** | **Exon**  **/Intron^b^** | **Prediction information^c^** | | | | | **AF in database** | | | **AF**  **in control**  **(n=200)** |
| --- | --- | --- | --- | --- | --- | --- | --- | --- | --- | --- | --- | --- | --- |
|  | **Nucleotide**  **(NM_016239.3)** | **Amino Acid**  **(NP_057323.3)** |  |  | **SIFT** | **Polyphen-2** | **Mutation Taster** | **GERP ++** | **Phylop** | **ExAC** | **1000**  **Genomes** | **gnomAD** |  |
| 1 | c.855dupT | p.Pro286Serfs*15 | frameshift | 2 | NA | NA | Disease Causing | NA | NA | 0 | 0 | 0 | A^d^ |
| 2 | c.3524dupA | p.Ser1176Valfs*14 | frameshift | 2 | NA | NA | Disease Causing | NA | NA | 0 | 0 | 0 | A |
| 3 | **c.3742C>T** | **p.Arg1248Trp** | missense | 4 | Damaging | Probably Damaging | Disease Causing | Conserved | Conserved | 0 | 0 | 0.000032 | A |
| 4 | c.4252G>A; | p.Gly1418Arg | missense | 11 | Damaging | Probably Damaging | Disease Causing | Conserved | Conserved | 0 | 0 | 0.000008 | A |
| 5 | c.4441T>C | p.Ser1481Pro | missense | 12 | Damaging | Probably Damaging | Disease Causing | Conserved | Conserved | 0.0001 | 0 | 0.000004 | A |
| 6 | c.4596+1G>A | Splice site | splicing | *13* | NA | NA | NA | NA | NA | 0.00002 | 0 | 0.000011 | A |
| 7 | **c.4597-2A>G** | Splice site | splicing | *13* | NA | NA | NA | NA | NA | 0.000008 | 0 | 0.000008 | A |
| 8 | **c.4666G>A** | **p.Ala1556Thr** | missense | 15 | Damaging | Probably Damaging | Disease Causing | Conserved | Conserved | 0.00003 | 0 | 0.00002 | A |
| 9 | **c.4747T>C** | **p.Ser1583Pro** | missense | 15 | Damaging | Probably Damaging | Disease Causing | Conserved | Conserved | 0 | 0 | 0 | A |
| 10 | **c.4823C>A** | **p.Ala1608Glu** | missense | 16 | Damaging | Probably Damaging | Disease Causing | Conserved | Conserved | 0 | 0 | 0 | A |
| 11 | **c.5507T>C** | **p.Leu1836Pro** | missense | 22 | Damaging | Probably Damaging | Disease Causing | Conserved | Conserved | 0 | 0 | 0 | A |
| 12 | **c.5692C>T** | **p.Arg1898*** | nonsense | 24 | NA | NA | NA | NA | NA | 0 | 0 | 0 | A |
| 13 | c.5964+3G>A | Splice site | splicing | *26* | NA | NA | NA | NA | NA | 0.000008 | 0 | 0.000029 | A |
| 14 | c.5977C>T | p.Arg1993Trp | missense | 27 | Damaging | Probably Damaging | Disease Causing | Conserved | Non-Conserved | 0.00002 | 0 | 0.00012 | A |
| 15 | **c.6177+1G>T** | **Splice site** | splicing | *28* | NA | NA | NA | Conserved | Conserved | 0 | 0 | 0 | A |
| 16 | **c.6479C>T** | **p.Pro2160Leu** | missense | 30 | Damaging | Probably Damaging | Disease Causing | Conserved | Conserved | 0.000129 | 0 | 0 | A |
| 17 | c.6796G>A | p.Val2266Met | missense | 33 | Damaging | Probably Damaging | Disease Causing | Conserved | Conserved | 0.0081 | 0.01 | 0.004802 | A |
| 18 | **c.7396-1G>A** | **Splice site** | splicing | *37* | NA | NA | NA | NA | NA | 0.000008 | 0 | 0.000014 | A |
| 19 | **c.7708_7709insCA** | **p.Gln2571Hisfs*35** | frameshift | 40 | NA | NA | NA | NA | NA | 0 | 0 | 0 | A |
| 20 | **c.7822G>A** | **p.Asp2608Asn** | missense | 41 | Tolerated | Probably Damaging | Disease Causing | Conserved | Conserved | 0.000017 | 0 | 0.000032 | A |
| 21 | **c.8033_8056del** | **p.Asn2678fs** | frameshift | 43 | NA | NA | NA | NA | NA | 0 | 0 | 0 | A |
| 22 | **c.8077del** | **p.Leu2693Cysfs*45** | frameshift | 43 | NA | NA | NA | NA | NA | 0 | 0 | 0 | A |
| 23 | c.8324G>A | p.Arg2775His | missense | 46 | Damaging | Probably Damaging | Disease Causing | Conserved | Conserved | 0.000008 | 0 | 0 | A |
| 24 | **c****.8771G>A** | **p.Arg2924His** | missense | 50 | Tolerated | Benign | Polymorphism | Non-conserved | Non-conserved | 0.000029 | 0 | 0.000039 | A |
| 25 | c.8791delT | p.Trp2931Glyfs*103 | frameshift | 51 | NA | NA | NA | NA | NA | 0 | 0 | 0 | A |
| 26 | c.10251_10253delCTT | p.Phe3420del | frameshift | 64 | NA | NA | NA | NA | NA | 0 | 0 | 0 | A |
| 27 | c.10420A>G | Ser3474Gly | missense | 65 | Tolerated | Probably Damaging | Disease Causing | Conserved | Conserved | 0.0005 | 0.002 | 0.0048 | A |
| 28 | **c.10502T>A** | **p.Leu3501Glu** | missense | 66 | Damaging | Probably Damaging | Disease Causing | Conserved | Conserved | 0 | 0 | 0 | A |

a. Nomenclature is based on NCBI accession number NM_016239.3，NP_057323.3 Novel variants reported in this study are in bold font

c. Missense variant pathogenicity Prediction Tools: SIFT, PolyPhen2 and Mutation Taster; Conservation tools: GERP++; PhyloP;

b. Location of *MYO15A* splicing variants in intron are showed in italic letters.

d. Letter A mean allele frequency of variant less than 0.01 in 200 ethnicity-matched normal hearing Chinese individuals.

Abbreviation in Table S2: NA, not available; AF, allele frequency of existing variant; Hom, homozygous mutation; Het, compound heterozygous mutation; ExAC, The Exome Aggregation Consortium; 1000 Genomes, 1000 Genomes database; gnomAD, The Genome Aggregation Database
